# Supplementary material for: Wind resource assessment Offshore Fujian using 30-year wind estimates
Source: PLoS One. 2025 Jun 25;20(6):e0326759. doi: 10.1371/journal.pone.0326759 (PMC12193848; doi:10.1371/journal.pone.0326759)
Supplement: Attachment — Submitted filename: Reply to Additional Editor Comments.docx [file pone.0326759.s002.docx]

Reply to Additional Editor Comments

First of all, the authors are very grateful to the editor to make the very constructive comments on our manuscript. We have realized that some places in our manuscript were unclear or improperly presented, not limited to the places mentioned below. We have taken full consideration of these comments and submitted a revised manuscript. In this point-by-point response, we reproduced the comments (black font), gave our responses (red font), and highlighted the revisions in new version of the paper in a red color.

We make the following major revision and reply to comment by the editor.

Some sentences are quite long and complex, which can make them difficult to follow. Breaking them up into shorter, more concise sentences would improve readability.

Reply: We revised the manuscript under the aid of professional scientific editor. And expect the revised version meet the publish standard.

The significance of choosing Fujian, China, as the study site isn't clear. Providing more context on why this location was selected would help readers understand its relevance.

Reply: In the “Section 2 Study area and observation data”, we have added the region description and selection season for the two particular observation station.

“Fujian’s coastal region, particularly the Taiwan Strait, is characterized by a “wind tunnel effect” due to its narrow topography, resulting in some of China’s strongest and most stable offshore wind resources. This area serves as a critical hub for China’s offshore wind energy development, with over 20% of the nation’s installed capacity concentrated here. The selection of two stations captures localized wind patterns influenced by monsoons, typhoons, and coastal-terrain interactions, which are underrepresented in existing global datasets.”

The exact locations of Xiapu and Pintan should be specified. Including geographical coordinates or a brief description of these areas would provide better context.

Reply: The exact locations of Xiapu and PingTan has been specified in Table 1.

Ensure consistency in the use of units throughout the paper. For example, wind speeds are given in m/s, and energy density in kWh/m²/year. Consistent use of units will make the data easier to compare and understand.

Reply: Yes, we use the consistency the units throughout the paper.

The methodology section could benefit from more detailed explanations. For instance, describe the types of wind measurement devices used and why they were chosen.

Reply: The Thies First Class sensors employed for these instruments are known for their high the anemometer and wind vane are recognized for their exceptional measurement accuracy and stability.

The introduction could be more engaging to capture the reader's interest. Consider starting with a compelling fact or statistic about the global energy crisis to draw readers interesting.

Reply: Yes, We revised the introduction according to your suggesting.

Explain why measurements were taken at specific heights (20, 50, 70, 80, 90, and 100 meters). This will help readers understand the rationale behind the data collection process.

Reply: The chosen heights comprehensively consider the balance between characterizing vertical wind profiles, equipment feasibility, and turbine design and energy yield validation. This approach ensures robust data for both theoretical modeling and real-world applications.
The term "AMSL" (above mean sea level) should be defined when first used to ensure all readers understand its meaning.

Reply: Yes, we have added the nomenclature in the introduction section following the reviewer’s suggestion.

Provide more context on the Measure-Correlate-Predict methodology. Explain how it works and why it was chosen for this study.

Reply: We have added “Section 3.8 Measure–Correlate–Predict method” in the analysis method section, and the new MCP method called Generalized Additive Model (GAM) is used in the revised manuscript. GAM address this limitation by integrating flexible smoothing functions, making them ideal for MCP analysis in spatially and temporally heterogeneous environments. Traditional MCP models poorly represent nonlinear interactions between mesoscale weather systems and local topography, leading to ±20% errors in extreme wind quantile estimation. GAM outperform linear models in capturing wind shear nonlinearities and turbulence-vegetation interactions, reducing prediction errors by 15–30% in coastal regions (Yang, 2021).

The transition between sections could be smoother. Adding transitional sentences or phrases would help guide readers through the paper more seamlessly.

Reply: Thank you for your good suggestion, we do our best to improve the writing.

Clarify the source and reliability of the ERA5 data. Discuss its accuracy and any potential limitations to give readers confidence in the data used.

Reply: Hourly 10 m and 100 m wind speed data from ERA5 is evaluated by comparing it with direct wind measurements obtained from 19 wind tower located across the coastal waters of China (Ji et al.,2025). They point out that the basic statistical characteristic between ERA5 reanalysis and observed wind speeds demonstrate good consistency. But, the ERA5 tends to underestimate wind speed, particularly at high speeds during extreme conditions (Ji et al.,2025).

The analysis of wind characteristics could be more detailed. For example, discuss the implications of average wind speed, wind direction frequency, and turbulence intensity on wind energy production.

Reply: We added the description hourly wind speed variation and mechanism analysis in the revised manuscript:

“Hourly wind speeds indicate that near-shore wind speeds in Fujian typically exhibit a bimodal variation. During morning surge (02:00-12:00), wind speeds increase by 15-20% compared to nighttime averages. During afternoon peak (14:00-20:00), maximum wind speeds occur, often exceeding 9 m/s above 70m height. Speeds gradually decrease after 00:00, with minimum values occurring before dawn. Wind speed differences between daytime peaks and nighttime lows average 2-3 m/s, reaching 4-5 m/s during winter monsoon periods. This diurnal signature is most pronounced in summer when thermal forcing dominates over synoptic systems. Such wind modal is related to Land-Sea thermal forcing. Solar heating intensifies land-sea temperature contrast during daytime, generating sea breezes that amplify wind speeds. The coastal terrain funnels these thermally-driven flows, creating acceleration zones. At night, reversed land breezes interact with background monsoon winds, causing partial speed reduction. Secondly, the Taiwan Strait's "wind tunnel" effect mechanically accelerates northwest monsoon winds. This effect peaks when solar heating strengthens pressure gradients across the strait. In addition, daytime convective mixing transports momentum from upper levels downward, enhancing surface winds. Stable nighttime atmospheric stratification inhibits this process.”

Explain the significance of the 30-year span in the context of the study. Why was this period chosen, and how does it enhance the reliability of the findings?

Reply: Meteorologists emphasize the need for at least 30 years of data to assess regional wind climates.

The suitability of different wind turbine classes needs more justification. Provide data or references to support the selection of specific turbine classes for the offshore site.

Reply: In the present study, the turbulence intensity, wind shear and extreme wind speed is analyzed, and is considerable three factors for wind turbine classes.

Discuss the implications of extreme wind speeds in more detail. How do these speeds affect the design and operation of wind turbines?

Reply: EWS during typhoons impose non-linear mechanical stresses on turbine components (e.g., blades, gearboxes, and towers). Typhoon-induced gusts exceeding 25 m/s can cause blade bending moments exceeding design limits, accelerating microcrack propagation. High turbulence intensity during typhoons increases gearbox vibration amplitudes by 30–50%, shortening lubrication cycles. These necessitates shorter inspection intervals (e.g., post-typhoon structural checks) and proactive replacement of fatigue-prone parts.

The recommendation for wind turbine class II should be supported with more data. Explain why this class is suitable based on the study's findings.

Reply: Wind turbine class II is recommended according to the extreme wind speed flowing the IEC recommendation.

Explain the criteria for upgrading to IEC Class A+. What factors were considered, and how do they impact the decision?

Reply: Wind turbine class Class A+ is recommended according to the extreme wind speed and turbulence intensity flowing the IEC recommendation.

The conclusion could summarize the key findings more effectively. Highlight the most important results and their implications for offshore wind energy development.

Reply: We have revised the “Section 5 summary and conclusions”, and highlighted the novel contributions.
Address any potential limitations of the study. Discuss how these limitations might affect the results and suggest areas for future research.

Reply: We have highlighted the key findings, and added the discuss the limitation of this study.

Consider the environmental impact of deploying wind turbines. Discuss any potential ecological effects and how they might be mitigated.

Reply: To ensure consistent energy production with lower wind speed period (e.g., in May), Some mitigation strategies should be implemented: 1) Technical optimization of wind turbines, deploy turbines with larger rotor diameters (e.g., 140–150m) and lower-rated capacities to capture low-speed wind energy more efficiently. For example, Goldwind's GW115/2.0 model increased annual output by 10.6% at 100m hub heights compared to 85 m towers in low-wind regions. Use hybrid steel-concrete or full-steel flexible towers (120–140m) to exploit higher wind speeds at elevated heights, consider the positive wind shear coefficients in the area. Optimize power tracking across low-to-medium wind speeds (2.5–5 m/s) through permanent magnet generators, achieving near-optimal power coefficient throughout the entire operational range. 2) Energy storage integration, deploy battery storage systems to store excess energy during high-wind periods and discharge during low-wind intervals, smoothing output fluctuations. Implement pumped hydro or hydrogen electrolysis systems for multi-day energy reserves. 3) Constructing multi-energy complementary systems, integrate photovoltaic panels to compensate for wind intermittency. For instance, solar generation typically peaks in summer, offsetting reduced wind output.”

The language could be more concise and precise. Avoid unnecessary jargon and ensure that technical terms are clearly defined. Ensure all technical terms are clearly defined. This will help readers who may not be familiar with the specific terminology used in the study.

Reply: Thank you for your good suggestion, we do our best to improve the writing.
The figures and tables should be referenced in the text. This will help readers understand how the visual data supports the study's findings.

Reply: Yes! Thank you for your suggestion.

The abstract should provide a clear overview of the study. Summarize the main objectives, methods, results, and conclusions in a concise manner.

Reply: Thank you for your good suggestion! The abstract has been rewritten carefully, and present the main findings of the study.

Proofread for any grammatical or typographical errors. Ensuring the paper is free of errors will enhance its professionalism and readability.

Reply: Reply: Thank you for your good suggestion, we do our best to improve the writing.
